# Thrombocytopenia: A possible side effect of apixaban

**DOI:** 10.1002/ccr3.2532

**Published:** 2019-11-21

**Authors:** Farid Sadaka

**Affiliations:** ^1^ Mercy Hospital St Louis St. Louis University St. Louis Missouri

**Keywords:** apixaban, newer oral anticoagulants, oral anticoagulant, platelets, thrombocytopenia

## Abstract

Apixaban is becoming more frequently prescribed for stroke prevention in patients with atrial fibrillation, and even rare side effects such as thrombocytopenia should be considered and monitored closely.

## Graphical abstract

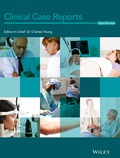

## 1 INTRODUCTION

Apixaban is one of the newer oral anticoagulants (NOAC). It works by the direct inhibition of factor Xa, which is formed by both intrinsic and extrinsic coagulation pathways. This prevention of thrombin formation from prothrombin is needed to prevent the conversion of fibrinogen to fibrin.^1^ Apixaban was approved by the FDA in 2011 for reduction in stroke and other thromboembolic events in nonvalvular atrial fibrillation.^2^ Apixaban has shown superiority in the prevention of stroke or systemic embolism versus warfarin with decreased risk of bleeding. As a result, apixaban is being increasingly prescribed. The trade name of apixaban is Eliquis (Bristol‐Myers Squibb). We report a case of thrombocytopenia that is possibly secondary to apixaban.

## 2 CASE REPORT

An 80‐year‐old male was admitted to the ICU with ventricular fibrillation episodes triggering multiple shocks by patient's implantable cardioverter‐defibrillator (ICD). Patient has a history of coronary artery disease (CAD) for which he underwent left heart catheterization (cath) in another hospital 1 month prior to this admission and was found to have severe right coronary artery (RCA) stenosis, but no intervention or stenting was done. He was diagnosed with cardiomyopathy for which he had biventricular ICD placed, and atrial fibrillation for which he was started on apixaban 2 months prior to this admission. He was also started on Amiodarone. Amiodarone was started and stopped after 2 weeks secondary to progressive shortness of breath and diagnosis of pneumonitis thought to be secondary to amiodarone toxicity. Last dose of amiodarone was about 2 months prior to this admission. His platelet level on admission was 109 × 10^9^/L, and it dropped to a nadir of 78 × 10^9^/L after 2 days of admission. Apixaban was stopped on admission in anticipation for left heart cath. He was also started on heparin drip, mexiletine, and carvedilol. Due to recent cardiac cath and use of heparin, anti‐heparin antibodies were sent for possible diagnosis of heparin‐induced thrombocytopenia (HIT), and heparin drip was switched to bivalirudin drip. Anti‐heparin antibodies were negative, thus ruling out HIT, and he underwent cardiac catheterization 3 days after admission with stent placed in the right coronary artery (RCA) and was placed back on heparin drip. On day 4, patient underwent ablation of AV node. Platelet levels started going up on day 4 reaching 137 × 10^9^/L on day 7. Patient had no clinical signs of thrombocytopenia (bleeding or bruising). On day 7, patient continued to deteriorate due to symptoms of congestive heart failure. He decided to proceed with comfort measures only, and patient passed away on day 8.

## 3 DISCUSSION

Thrombocytopenia secondary to apixaban has been reported by the drug's package insert (<1%)^3^; however, only one previous case was reported with possible apixaban‐induced thrombocytopenia.^4^ Thrombocytopenia in our case seems to be most probably secondary to apixaban; however, other possibilities should be considered, especially other medications. Thrombocytopenia has been reported rarely with amiodarone, however, unlikely after 2 months of stopping the medication. Mexiletine could cause thrombocytopenia; however, thrombocytopenia was present on admission prior to starting mexiletine and started improving despite patient remaining on it. Heparin should always be considered as a probable culprit for thrombocytopenia; however, in this case, anti‐heparin antibodies were negative, and platelet levels continued to improve despite switching back from bivalirudin to heparin drip. In addition, the timeline coincided with apixaban, since it was started 2 months prior to admission, and apixaban was stopped on admission, followed by gradual improvement in platelet levels. The other common diagnosis frequently associated with thrombocytopenia, sepsis, was not diagnosed or treated in this patient. In addition, there was no evidence of DIC, TTP, anemia, or neutropenia on laboratories. Thrombocytopenia could not have been secondary to cardiac catheterization or devices (ICD, stent) because of the timing. Platelet levels improved after cardiac cath, and ICD was placed 2 months prior to admission and was not removed. Possible mechanisms of apixaban‐induced thrombocytopenia include immune‐mediated reaction^5^ via drug‐dependent antibodies, or bone marrow suppression.^6^ The rapidity of improvement in platelet number favors an immune‐mediated hypothesis, since bone marrow recovery typically needs more than 7 days.

Apixaban is becoming more frequently prescribed for stroke prevention in patients with atrial fibrillation, and side effects such as thrombocytopenia should be considered when patient is on apixaban. This is only the second case report to our knowledge that has strongly documented this association. It is possible that thrombocytopenia in both reports may not be related to apixaban; however, since it is a relatively new and its prescription is rapidly increasing, even uncommon side effects need to be monitored closely and reported. This is especially important now with increased use of NOAC for treatment of HIT.^7^

## AUTHOR CONTRIBUTIONS

FS and MD: solely contributed to the conception and design of the study, the acquisition, analysis, interpretation of data, the drafting of the manuscript, revising it critically for important intellectual content, and final approval of the submitted manuscript.
